# Advances in Microbial Biofilm Prevention on Indwelling Medical Devices with Emphasis on Usage of Acoustic Energy

**DOI:** 10.3390/s90402538

**Published:** 2009-04-14

**Authors:** Naama Dror, Mathilda Mandel, Zadik Hazan, Gad Lavie

**Affiliations:** 1 Department of Cellular and Developmental Biology, Tel-Aviv University, Tel-Aviv, Israel; E-mail: dror.naama@gmail.com (N.D); 2 Blood Center, Sheba Medical Center, Tel-Hashomer, Israel; E-mail: mandel@sheba.health.gov.il (M.M); 3 Regenera Pharma Ltd., Rehovot, Israel; E-mail: zadikster@gmail.com (Z.H)

**Keywords:** Anti-microbial agents, Biofilms, Biofilm prevention, Acoustic energy, Ultrasonication

## Abstract

Microbial biofilms are a major impediment to the use of indwelling medical devices, generating device-related infections with high morbidity and mortality. Major efforts directed towards preventing and eradicating the biofilm problem face difficulties because biofilms protect themselves very effectively by producing a polysaccharide coating, reducing biofilm sensitivity to antimicrobial agents. Techniques applied to combating biofilms have been primarily chemical. These have met with partial and limited success rates, leading to current trends of eradicating biofilms through physico-mechanical strategies. Here we review the different approaches that have been developed to control biofilm formation and removal, focusing on the utilization of acoustic energy to achieve these objectives.

## 1. Introduction

Indwelling medical devices have become major tools in the clinical management of hospitalized patients, particularly those requiring life supporting devices. Urinary, intratracheal, central vein, peritoneal dialysis nephrostomes and other indwelling devices are becoming increasingly frequent in medical practice and are applied to more than 25% of hospitalized patients. However, as the duration of their placements become prolonged, risk factors related to microbial infections and biofilm formation culminate in higher morbidity and mortality rates among hospitalized patients, and sending the costs of hospitalization spiraling upwards. Most circulatory and urinary tract infection cases are associated with indwelling medical devices [1]. Microbial biofilms develop on the surfaces of medical devices and proceed to cause full blown bacterial infections and sepsis. In patients with urinary catheters, infection rates increase with the duration of catheterization at rates of 5–10% per day with virtually all of those who undergo long-term catheterization (>28 days) becoming infected [2–4]. Estimates by Costerton attribute more than half of bacterial infections in immuno-compromised patients to slime encased microbial colonies (biofilms) [5]. The U.S. National Institutes of Health mention infection rates as high as 80% that are due to microbial biofilms [6].

The magnitude of the biofilm problem and its impact on medical and financial aspects of modern hospital medicine have triggered the investment of major efforts to develop novel anti-biofilm strategies based on thorough, in depth analyses of the microbial transformation cascades. The rationale has been to develop means for disruption of colony formation at multiple sites of these transformation cascades and for eradication of existing biofilms. The composition of inert surfaces and intersurfaces has come under review as these form substrates to which unicellular planktonic microorganisms attach to enable their transformation into the multicellular sessile forms.

The encasing slimy exopolysaccharide matrix which is secreted by the microorganisms and in which developing colonies become encapsulated, is another major target. This unique architecture regulates exchange of ions, chemicals and nutrients with the surrounding environment. It thus protects biofilms from external insults by blocking entry of biocides, surfactants and predators and renders them 1,000 times more resistant to antibiotics compared to free floating bacteria. [7,8]. In addition to acting as transport barriers to agents harmful to biofilms [9], exopolysaccharide matrix polymers also bind to and neutralize antibiotics prior to their interaction with bacteria [10]. Even if the antibiotics are successful in penetrating into the biofilm other researchers suggest that bacteria within biofilms are dormant and do not actively metabolize antibiotics [11].

## 2. Biofilms and Anti-Microbial Immunity

Microbial cells within biofilm colonies are also much less susceptible to host immune mechanisms. Key antigens are either repressed or concealed from effector immune cells [12], and bacteria in colonies are highly resistant to phagocytosis by immune system phagocytes [12]. Deposition of complement C3b and IgG on bacterial surfaces has also been shown to be prevented as demonstrated for *Staphylococcus epidermidis* [13], contributing to protection of bacteria from killing by polymorphonuclear leukocytes. Furthermore, in airways of cystic fibrosis patients the presence of polymorphonuclear leukocytes has even been found to enhance *Pseudomonas auruginosa* biofilm formation due to bacterial binding to F-actin and DNA polymers [14]. Thus, the various arms of anti-microbial immunity are neutralized by the biofilm exopolysaccharide protective matrix, leaving affected patients fully vulnerable to the problem.

Of interest are results of studies which have evaluated the effects of effector molecules of innate immune mechanisms on formation and survival of various types of microbial biofilms. Molecules such as lactoferrin, a constituent of human external secretions, have been found to inhibit development of *Pseudomonas aeruginosa* biofilms at lactoferrin concentrations lower than those that kill or prevent growth of the planktonic cells. The authors suggest that by chelating iron lactoferrin stimulates twitching surface motility, causing dispersion of bacteria rather than formation of the cell clusters required to form biofilms [15]. The importance of these observations is in the principle which suggests existence of a specific anti-biofilm formation protective mechanism. It acts at the stage in which bacteria begin to aggregate to form communities that subsequently transform into the sessile form of life [15]. Lactoferrin, however did not prevent fungal biofilm formation. This could be partially achieved by using oxidative and non-oxidative antimicrobial molecules produced by phagocytic cells such as PG-1, β-defensin-1, and β-defensin-3, which significantly reduce the metabolic activity in the biofilm [16]. Altogether immune reactions do not effectively inactivate biofilms but rather further invigorate the unique and highly resistant properties of microbial biofilms. The compromise of anti-microbial immune mechanisms is the basis for the difficulties in eradicating this major source of microbial infection, explaining the severity, persistence and high morbidity associated with biofilm-derived infections.

## 3. Chemical Approaches, To Biofilm Elimination

Chemical modifications constitute the main strategy being contemplated for biofilm elimination on indwelling medical devices. In one such approach catheters are coated with antimicrobial agents such as antibiotics, biocides such as chlorohexidine or with ion coatings with silver and to a lesser extent with zinc. These interfere with the attachment and expansion of immature biofilms. According to Riley, who conducted the largest randomized clinical trial with silver impregnated urinary catheters in 1,309 patients, the silver coating was not found to be efficacious in preventing bacteriuria and on the contrary, increased incidence of staphylococcal bacteriuria has been reported to occur with silver coated catheters [17]. Unlike the findings with urinary catheters, evaluation of silver coatings on endotracheal tubes aimed at preventing ventilator-associated pneumonia (VAP) yields far more promising results. This study, conducted in a large single-blind, controlled study by the NASCENT Investigation group, enrolled 9,417 adult patients in 54 centers in North America. The incidence of VAP based on quantitative cultures from bronchoalveolar lavage fluids, has been evaluated. VAP was found to decline from 7.5% per ≥ 24 hrs of intubation with uncoated endotracheal tubes to 4.8% in the group receiving the silver-coated tubes [18]. The study concluded that patients who received silver-coated endotracheal tubes had statistically significant reductions in VAP incidence and experienced delays in the time to VAP occurrence compared with patients who received similar, uncoated tubes [18]. Another important conclusion from the urinary versus endotracheal catheter studies is that different results may be obtained for different types of catheters and each catheter type should be evaluated separately irrespective of results obtained with other catheter types.

In patients requiring prolonged intravenous catheterization, microbial colonization of the intraluminal surfaces, generates biofilms that cause serious catheter-related bloodstream infections. To prevent these infections and salvage the central vein catheters an intraluminal antimicrobial lock therapy has been developed. The lock therapy involves sequestration of 2 to 4 mL of biocides in the catheter at concentrations 100–1,000-fold higher than the maximal dose that can be used systemically. The solution is allowed to dwell (lock) when the catheter is not in use, eradicating the bacteria and fungi embedded in the intraluminal biofilms [19]. Combinations of vancomycin and heparin have been frequently used in antimicrobial lock therapy however, these met with only partial success [20,21]. Use of minocycline and EDTA in lock times of 4 hrs, have proven to be more effective against bacterial biofilms [22]. Supplementation with the antifungals echinocandins- Caspofungin and micafungin, (1,3)-beta-D-glucan synthase inhibitors have been suggested to expand the effective range to also include fungal colonies [23].

Another implant setting in which biofilms formed by *Staphylococcus epidermidis* cause pathologic reactions, is found in silicone breast implants. The problem of capsular contracture formation is traditionally addressed by systemic administration of antibiotics combined with antiseptic washings. Yet, a recently developed *in vitro* model for silicone breast implants suggests that combinations of chloramphenicol (Chloramex^®^), sodium fusidate (Fucidin^®^) and terramycin are likely to yield better results [24]. This model has yet to receive clinical confirmation.

Interference with the attachment or adhesion of microorganisms to solid substrates, which is the first essential step of colonization, is a strategy that has been used for quite some time in the struggle against microbial biofilm formation. Bacterial adhesion is a broad and heterogeneous phenomenon as bacteria attach to a range of substrates, from indwelling catheters to epithelial tissues of the skin and GI, urinary and respiratory tracts. Adhesion is mediated by lectin-like, carbohydrate binding molecules known as adhesins. Several organelles have evolved to mediate the adhesion process including pili [25,26], fimbriae [27], flagellae [28] and outer membrane proteins [29]. They interact with carbohydrates present on cells and other surfaces in specific manners. Initial efforts have been to interfere with microbial attachment to surfaces via specific interventions that block carbohydrate binding sites. Mannosides and sialic acid compounds were used to target type I and type IV pili, respectively, however carbohydrate specific intervention strategies could not address the microbial adhesion problem at large. Multiple adhesion mechanisms have evolved on bacteria including non specific forms of adhesion, which do not appear to involve specific adhesins. For example when bacteria reach very close proximity to solid surfaces, at distances of 2 to 50 nm they are found within the range of van der Waals, hydrophobic, ionic, and electrostatic interaction forces [30]. Adhesion is initiated, mediating steps essential for promoting bacterial invasion and pathogenesis. In addition, surface binding mechanisms used by different microorganisms, are heterogeneous. *Pseudomonas aeruginosa* pili bind to asialoGM1 [31], whereas *E. coli* pili target mannosides and *E. coli* fimbriae N-acetyl glucose amine [32]. This heterogeneity is also supplemented by additional mechanisms such as secretion of enzymes as neuraminidase, which remove surface sialylated sugars. Microbial binding targets are modulated exposing cryptic binding ligands [33,34]. These further complicate development of strategies for both prevention of biofilm formation and eradication of existing biofilms.

A different and more successful prevention strategy of microbial adhesion and biofilm development, targets the surfaces of devices to reduce their compatibility with microbial cell attachment and improve their anti-microbial cytotoxicity. A broad range of chemical modifications of device surfaces have been developed to form unique contact-killing surfaces and biomaterials that are incompatible with adhesion. All-silicone, silicone-coated latex and nitrofurazone silicone catheters have been used in some cases in combination with hydrogel, hydrogel/silver-coatings of latex or silicone-based, silver-impregnated catheters. These strategies have been evaluated for prevention of nosocomial urinary tract infections and found to be ineffective in preventing biofilm formation. Use of silver alloy coated Foley catheters rather than silver ion coated ones did reduce the frequency of catheter acquired urinary tract infection with effects that exceeded one week of catheterization, however overall the effects were mainly short lived and declined after catheterization intervals of one week [35,36].

The most effective biofilm prevention method reported thus far has been achieved by coating of ureter polyurethane stents with heparin. The coating was shown to provide effective prevention of biofilm development compared with uncoated stents *in vivo.* Inhibitory effects persisted for 6 – 8 weeks of indwelling [37].

The coating of indwelling catheters with various types of antibiotics or other disinfectants is associated with their leaching over time from the catheter surfaces to surrounding tissues. The leaching leads to reduced concentration of the anti-microbial agent on the catheter surface rendering any efficacy temporary and creating optimal conditions for development of resistant microbial strains. Recently, a novel concept in modification of device surfaces has been successfully developed. The new technology involves stable immobilization of anti-microbial agents on device surfaces via long and flexible polymeric chains covalently anchored to the device surface. Non-leaching contact-killing surfaces are produced. The developers used the quaternary ammonium derivative - *N*-alkylpyridinium bromide as the antimicrobial agent which was attached to a poly(4-vinyl-*N*-hexylpyridine) used as the anchoring carrier of the polymer [38]. The polymer was capable of inactivating ≥99% of *S. epidermidis*, *E. coli*, and *P. aeruginosa* bacteria on glass slides processed as non-leaching contact-killing surfaces *in vitro*. In their quest to characterize the structural requirements from polymers to be active as bactericidal agents the authors modulated the hydrophobicity and the positive charge by using several other backbone compounds and antimicrobial moieties. These studies revealed the importance of positively charged polycationic chains in enabling the molecule to stretch out and generate bactericidal activity. A study in which sterile surfaces were created on paper or glass by forming a poly[2-(dimethylamino)ethyl methacrylate] polymer, was used to examine the question of whether the sterile surface kills bacteria or only inhibits their growth. The immobilized polymers which were sufficiently long for traversing the approximately 30 nm cell envelope were found to be cytotoxic and killed bacteria as well as fungi via mechanisms that were not susceptible to development of resistance. Most importantly, silicone rubber to which 3-(trimethoxysilyl)propyldimethyloctadecyl ammonium chloride was covalently coupled showed antimicrobial properties against adhering bacteria, both in vitro and in vivo. Rats in which the surface modified silicone rubber was implanted subcutaneously for three or seven days had infection at rates of seven out of eight silicone rubber implanted animals decline to one out of eight when the silicone rubber implants were coated with positively charged polycationic chains [39].

In addition to existence of non specific adhesion mechanisms, the general lack of antimicrobial efficacy of different chemical modification strategies are also thought to result from conditioning films which develop on the catheter surfaces. Such films arise as a result of incrustations with proteins, electrolytes and other organic molecules on indwelling devices. The incrustations develop shortly after device insertion, modify the outer surface of the device and conceal its antimicrobial agents, enabling in this way the formation of biofilms [40]. It will also be of interest to find out if non-leaching contact-killing surfaces can maintain long term microbicidal activity on indwelling catheters in light of the “conditioning film” problem. Lubrication of catheter surfaces, have attempted to limit such encrustations. Hydrogel as a single lubricant or in combination with silver coating, have been examined, however as mentioned above, these were also ineffective in preventing bacterial biofilm formation on urinary catheters [41].

Other factors that contribute to the overall failure of chemical approaches to prevent biofilm formation on indwelling medical devices include increased resistance of bacteria in biofilms to antimicrobial agents. Increased resistance to antimicrobials could be due to markedly down-regulated metabolism in bacteria within biofilms [5]. Indeed, adhesion of bacteria to solid surfaces has been shown to lead to diminution in the number of genes that are expressed compared to planktonic bacteria. Factors repressing a large number of genes such as the σ factor render biofilm bacteria phenotypically different from planktonic ones [42]. This adds to the interference with the transport of antibiotics and biocides into biofilms by the slimy exopolysaccharide encasing [43,44], rendering the sessile colony lifestyle highly resistant to eradication to this very day.

## 4. Biological Approaches to Bacterial Biofilm Eradication

A novel and interesting approach that has recently emerged is to treat biofilms with lytic bacteriophages. The attractive element in this concept is the finding that phages produce polysaccharide lyases that act as endo-glycanohydrolases. These enzymes are depolymerases capable of degrading biofilm exopolysaccharide matrix polymer components. Phages can then penetrate the inner layers of the biofilm, infect bacteria in the colony and cause their lysis [45]. This approach is being used to treat *Pseudomonas aeruginosa* biofilms in cystic fibrosis patients by spreading aerosol that contains the phages [46]. Phages can also be engineered to express the genes encoding these polysaccharide lyases used to elicit the biofilm exopolysaccharide dissolution to possibly, expand the number of bacterial species that can be targeted by a phage [47].

## 5. Interference with Inter-Bacterial Signaling for Biofilm Formation

Following attachment to solid surfaces bacteria secrete several classes of small, diffusible quorum sensing molecules also known as autoinducers. These molecules form concentration gradients that convey inter-bacterial signaling capable of modulating bacterial gene expression to patterns that transform the planktonic lifestyle into a sessile form [48–50]. Their activity promotes biofilm development and differentiation. Oligopeptides and *N*-acylhomoserine lactones are involved in group-specific communications within gram-positive and gram-negative bacteria respectively, and boronated-diester molecules in communication among both gram-positive and gram-negative bacteria [51–54]. Disruption of autoinducer signaling pathways have been hypothesized to help prevent biofilm formation and differentiation. Two approaches have recently been proposed for interfering with these biofilm differentiation-promoting signaling mechanisms: one is biological and the other is mechanical. The biological approach identified naturally occurring products such as furocoumarins, found in grapefruit juice, that are capable of inhibiting cell-to-cell autoinducer signaling between bacteria, inhibiting biofilm formation [55]. The authors reported >95% inhibition of all types of autoinducers, gram negative or gram positive specific, as well as interspecific classes. The efficacy of these compounds, were analyzed in a *Vibrio harveyi* based autoinducer bioassay and in biofilm formation assays by *Escherichia coli*, *Salmonella typhimurium* and *Pseudomonas aeruginosa*. Although they appear to be effective *in vitro*, these products have yet to prove their effectiveness *in vivo;* their routes of administration must also be delineated more precisely, as grapefruit juice consumption is abundant and not known to prevent life threatening infections due to bacterial biofilms.

Quorum sensing signaling in bacteria has also been tackled through mechanical approaches aimed at disruption of the concentration gradients of the small molecule mediators. Ultrasonic acoustic energy is being investigated as a mean for disrupting quorum sensing signaling through induction of chaos in the concentration gradients. The aim is to prevent bacterial cell migration and assembly at sites of colony formation, interfering in this way with biofilm differentiation [51,56] as well as with induction of several additional anti-biofilm effects that are discussed later in this review.

## 6. Uses of Acoustic Energy in Biofilm Prevention

The inability to eradicate microbial biofilms by chemical means raises the prospect that mechanical strategies may deliver better outcomes. A favored mechanical approach has been the use of acoustic energy for preventing *de novo* formation of biofilms as well as for disrupting the integrity of existing ones. Multiple objectives were expected to be achieved with the use of ultrasonic energy:

1. Abrogation of the two initial steps in biofilm formation: adhesion of planktonic microorganisms to surfaces and the ensuing firm attachment to substrates [57].
2. Creation of stable cavitation in biofilms to enable more effective penetration and transport of antimicrobials and biocides through their exopolysaccharide encasings.
3. Bypassing the obstacle of conditioning-films, which interfere with microbicidal coatings from reaching biofilms.
4. Creation of microstreamings, which disrupt autoinducer gradients and abolish its signaling. Another byproduct of microstreamings is enhancement of the rate of cellular metabolism. Ultrasound is presumed to improve oxygen and nutrient transport to cells within biofilms and to planktonic cells [54,58], resulting in acceleration in the metabolic rates of the cells. The elevated metabolic rates may render bacterial cells more susceptible to antibiotics [58]. The hypothesis is that by invigorating the rate of bacterial cell metabolism ultrasound improves the efficiency of antibiotics.
5. Infliction of mechanical damage to existing biofilms.

Indeed, in studies performed in *E. coli* and *Pseudomonas aeruginosa, in vitro* ultrasonication has been found to significantly increase transport of antibiotics (gentamicin) across biofilms*,* enhancing the killing of bacteria within the biofilm encasing [59]. However, in the absence of the antibiotic these same ultrasonic parameters (continuous ultrasonication at a frequency of 500 kHz and power density of 10 mW/cm^2^, *in vitro*), did not alter neither the *Pseudomonas aeruginosa* biofilm structure, nor cell organization within these biofilms. Confocal scanning laser microscopic (CSLM) analyses revealed that these structures remained intact [60].

*In vivo*, the susceptibility of biofilms produced by different types of bacteria to ultrasound-enhanced transport of antibiotics is differential. Viability of *E. coli* biofilms formed on implants has been found to decrease significantly following simultaneous administration of gentamicin with pulsed low frequency ultrasound (1:3 duty cycles applied at power intensity of 500, mW/cm^2^ for 24 and 48 hrs). Such reductions have not been observed in experiments conducted with *Pseudomonas aeruginosa* biofilms [61–63]. The authors offer several explanations for the observed differences between treatment susceptibilities of the two types of biofilms *in vivo.* (1) Existence of differences in permeability of the outer membranes of the two bacteria [60–62,64–66], (2) higher efficacy of aminoglicosides against metabolically active *E. coli* bacteria which may be more active in this model [67], and (3) oxygen limitations in the implant environment that may render *E. coli* biofilms more susceptible to antibiotics.

These findings were confirmed by Ensing and co-workers [68]. They have shown that application of ultrasound at frequencies of 28–48 kHz and maximal intensity of 500 mW/cm^2^ combined with gentamicin administered systemically or released from antibiotic-loaded bone cement, reduced bacterial viability in *E. coli* biofilms by >50% [68]. Combinations of pulsed ultrasound with an antibiotic released slowly from bone cements (preloaded with antibiotics) were found to significantly reduce the viability of both planktonic bacteria and biofilms from clinical isolates, compared to antibiotics released in the absence of acoustic energy [69]. Here too, the effect could not be produced successfully in *P. aeruginosa*.

The selectivity in responsiveness to combinations of ultrasound and antibiotics, with some bacteria being affected whereas others are not renders importance to evaluation of the responsiveness of *Staphylococcus epidermidis.* Gram positive bacteria such as *S. epidermidis* are commonly found in infections affecting orthopedic and other types of implants [70]. These bacteria were subjected to combination treatments with low frequency ultrasound and systemic vancomycin for 48 hrs in a rabbit model. The viable bacterial counts were reduced significantly by the combined treatment with ultrasound and antibiotics relative to antibiotic treatment alone. Prolongation of the duration of the combination treatment also appeared to enhance the biocidal effects [71].

The partial and incomplete reductions in bacterial counts within biofilms, obtained with combined treatment with acoustic energy and antibiotics, prompted search for additional improvements in biofilm eradication efficacies. These were sought by further adding proteolytic enzymes to treatments applied to solid surfaces. Oulahal-Lagsir and co-workers applied treatments with 40 kHz of ultrasonic energy for durations in the range of 10 seconds combined with applications of protease for 15 minutes or trypsin for 30 min. These combinations yielded synergistic effects of higher efficacies, removing 84-95% of *E. coli* biofilms compared to 30% with acoustic energy alone [72]. Although initially developed for the food industry, these combinations may form a basis for modifications that will involve endogenous human proteases for use on medical devices.

## 7. Complexity of High Frequency Acoustic Energy Effects on Biofilms and the Need for Improved Understanding of the Phenomenon

The high potential embedded in the use of ultrasonic acoustic energy for preventing, suppressing and disrupting many aspects of biofilm life attracted extensive research efforts aimed at bringing it to clinical use. Since various laboratories used different systems, contradictory findings soon emerged. In contrast to the biocidal effects of combinations of antibiotics and ultrasound, Pitt and colleges found that low intensity ultrasound (2 W/cm^2^) administered at a low frequency of 70 kHz as a sole treatment, enhances growth of both *S. epidermidis*, *E. coli* and *P. aeruginosa* biofilms as well as their planktonic forms. This was presumed to result from improved oxygen and nutrient transport to cells within ultrasound treated biofilms and planktonic cells [58].

The conflicting results obtained when parameters of acoustic energy used in anti-biofilm microbiology were varied revealed the complexity of the system. It also highlighted the variability associated with the type of endpoints which were sought – biofilm prevention versus biofilm eradication. Each appears to require for optimal activity very different types of acoustic energy, varying in both frequency and intensity. Thus, the levels of ultrasonic energy that are applied may play crucial roles in the outcomes of treating existing biofilms or in preventing their de novo formation.

For example, high ultrasonic power density levels were found to be highly effective in stripping surfaces from existing biofilms. Surfaces covered by 10^9^ CFU/mL of bacteria could be cleaned by axially propagated ultrasound (APU) [71]. APU applied as 30 second pulses at intensities of 35–45 Watts or 6–9 W, using probes suitable for frequencies of 350 kHz, 150 kHz and 20 kHz, respectively, effectively removed *Proteus mirabilis* biofilms from water-filled glass tubes. However, here too efficacy was inversely related to the frequency used. Treatment with 20 kHz proved the most efficient, removing 87.5% of the biofilm from 50 cm tubes, whereas APU applied at 150 kHz and 350 kHz removed only 66.8% and 31.3% of biofilms, respectively [72].

The complexity of factors involved in the use of acoustic energy in biofilm biology, become all the more evident when prevention of de novo formation of biofilms is sought. Unlike eradication, prevention of biofilm formation involves entirely different mechanisms such as interference with adhesion to solid surfaces, with formation of quorum sensing molecular gradients that affect biofilm differentiation and others. The acoustic energy properties required to effectively interfere with these mechanisms prove to be unique for each of the indications.

In a set of elegant studies Pitt and colleagues analyzed and defined the requirements for effectively interfering with biofilm formation using acoustic energy. They postulated that when bacterial vibration amplitudes are smaller than those of surfaces located at close vicinity, a relative velocity of bacteria respective to the surface occurs as governed by Stoke’s law [58]. When the bacterial vibration amplitudes are smaller than the Z (Zeta) potential, a repulsive zone develops in the vicinity of a solid surface and an overall net repulsion occurs that prevents bacterial attachment. Increasing the bacterial vibration amplitudes to values exceeding the Z potential repulsion zone, results in a net attraction force that promotes the adhesion of bacteria [73,74] yielding opposite results. Systems that conform to such conditions have been found to effectively prevent the initial steps in biofilm formation - the adhesion and firm attachment of microorganisms to the catheter surfaces. These systems include the one which we have developed, which is discussed hereunder.

## 8. Fine Tuning the Acoustic Energy Properties is Required for Each Type of Indwelling Catheter

Elaborate fine tuning of the vibration energy properties is emerging as a requirement for obtaining effective prevention of biofilm formation on different types of indwelling medical devices. We noted this requirement when we devised an innovative approach to prevent biofilm formation on catheters. It involved development of small piezoelectric elements which we attached to solid surfaces and to extracorporeal sections of catheters. The piezo elements generate low energy acoustic waves and this energy is delivered directly to the catheter. The waves spread throughout the catheter and its surfaces, covering the catheter with a vibrating coat that is also dispersed throughout the surrounding fluid media, causing the bacteria to vibrate with the same frequency [75]. Conditioning films encrusted with proteins, electrolytes and other organic molecules which are known to develop on urinary catheters shortly after their insertion [36,40] do not appear to interfere with biofilm prevention by the low energy acoustic waves [75].

**Figure 1.**
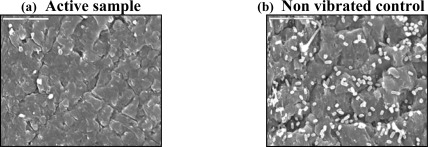
Effects of low levels of acoustic energy on bacterial attachment and biofilm formation on a urinary catheter. Scanning electron microscopic analysis of a French 8 urinary catheter (a) connected to a Piezo electric element emitting 0.2 mW/cm^2^ of vibration energy, and (b) catheter with a non vibrating element. The catheter was immersed in a suspension of *E. coli* bacteria for 48 hours.

Our findings show that certain power intensities (Po_int_ ≤ 1.1 mW/cm^2^ and frequencies of 100–300 kHz) appear to constitute the optimal acoustic energy levels for preventing biofilm formation on urinary catheters. This type of low acoustic energy inhibits bacterial attachment and biofilm formation on catheter surfaces significantly as evident from scanning electron microscopic (SEM) analyses as shown in Figure 1 and discussed in [75].

In studies we conducted *in vitro* on four types of microbes, *E. coli*, *Enterococcus faecalis*, *Candida albicans* and *Proteus mirabilis* SEM analyses revealed that the piezo element-generated acoustic waves reduced the viable microbial counts on the catheter surfaces significantly, and effectively diminished biofilm formation [75]. Catheters remained virtually clean of adherent microorganisms, indicating that this technology is efficacious against a broad spectrum of microorganisms and not limited to selected groups. The surface acoustic waves create chaotic microstreaming, which interferes with bacterial attachment as well as with post attachment biofilm developmental processes.

Increasing the acoustic energy power intensity levels or the wave frequencies resulted in a dramatic shift from inhibition to strong enhancement of biofilm formation. These higher acoustic energy intensities activate bacterial docking and force sensor activities and this synergism can elicit the increased adhesion of bacteria [75].

A model which shows this shift from inhibition to strong enhancement in bacterial adhesion with increased acoustic energy output is the mannose receptor-mediated adhesion of *E. coli* to guinea pig erythrocytes. This specific adhesion occurs via *E. coli* FimH lectin binding to mannosides on surfaces of guinea pig erythrocytes. Adhesion of the bacteria to erythrocytes, causing their aggregation is effectively inhibited by low acoustic energy levels. In fact the optimal range of acoustic energy that inhibits *E. coli* adhesion to the red blood cells is even lower than the levels that inhibit adhesion to catheters (ranging between 0.05–0.20 mW/cm^2^). In contrast, increasing the piezo element-generated acoustic energy to > 0.35 mW/cm^2^ again induced opposite effects with strong bacteria-mediated RBC adhesion and enhanced aggregation [75].

The most important findings in these studies were obtained in a rabbit model *in vivo*. Indwelling urinary catheters inserted into the meatus of male rabbits and vibrated via the attached actuators enabled the maintenance of catheter sterility for 7.3 ± 1.3 days (up to 9 days), whereas, control rabbits developed bacteriuria of >10^5^ CFU/ml within 1.5 ± 0.6 days [75].

The sharp transition to opposite effects on biofilm development that occur in the presence of different acoustic energy dose/frequency levels, reveal a major weakness in utilization of piezo-element generated acoustic energy for preventing biofilm formation on indwelling catheters. The problem stems from difficulties in precisely controlling the acoustic energy output from the piezo actuators. Somewhat higher outputs enhance bacterial attachment to the catheters and promote biofilm differentiation rather than inhibit them. Thus, the application of surface acoustic waves to various types of medical devices requires meticulous adjustments of frequencies and amplitudes for each type of targeted catheter.

## 9. High Energy Ultrasound

Ultrasonic energy used in the combat against microbial biofilms is divided into two categories with respect to the effects it produces: power intensities that cause cavitation are energy levels in excess of the cavitation producing energy thresholds. These are wave frequencies f ≥ 100 kHz generated at acoustic power intensities of 0.5–2×10^3^ mW/cm^2^. A second category includes the lower power intensities that do not form cavitations [76]. The high acoustic energy power intensity levels are more suitable for eradication of existing biofilms rather than preventing their formation.

Biofilm removal was found to strongly depend on the intensity of acoustic energy and to a far lesser extent on the frequency [77]. The author reported that coupling the acoustic energy with convective fluid flow dramatically improved biofilm removal at acoustic intensity of 27 W/cm^2^ (removal of up to 80% of biomass in two minutes and close to 100% when intense ultrasonication was coupled with gas bubbles in the fluid) [77, 78]. Unlike these power intensity doses prevention of biofilm formation is effective upon transmission of much lower acoustic energy intensity levels (≤ 0.35 mW/cm^2^) [75].

## 10. Future Outlook and Conclusions

Despite significant breakthroughs in biofilm prevention and eradication technologies, current relief is only short lived, limited to certain types of catheters and unsuitable for others. In many cases efficacy is seen on some types of bacteria and not on others. The likelihood for broadening the spectrum of chemical anti-biofilm specific reagents may depend on the identification of the entire spectrum of saccharides, proteins, mucins and lipids that the various bacteria can target for microbial adhesion. One would then be required to develop combinations of inhibitors that can counteract each of the receptors.

The current outlook for the general and non-specific anti-biofilm arena is more likely to employ mechanical means in the form of various types of acoustic energy. Arrays of modifications and adjustments that will render the low acoustic energy conditions suitable to different indwelling medical devices, are likely to expand its scope of use and may render this technology suitable for utilization in central vein catheters and possibly also in endotracheal as well as other catheters. There is also the potential of utilizing acoustic energy to prevent biofilm formation on organs and not only on devices. One example which we have begun to evaluate is prevention of intra-tracheal biofilm formation, a utilization that may be of importance for patients with cystic fibrosis. Preliminary studies on sheep trachea reveal the complexity of applying this concept even before addressing the problem of the high intolerance of the airways to foreign bodies.
